# Electrochemical Dopamine Biosensor Based on Poly(3-aminobenzylamine) Layer-by-Layer Self-Assembled Multilayer Thin Film

**DOI:** 10.3390/polym13091488

**Published:** 2021-05-06

**Authors:** Tayanee Panapimonlawat, Sukon Phanichphant, Saengrawee Sriwichai

**Affiliations:** 1 Department of Chemistry, Faculty of Science, Chiang Mai University, Chiang Mai 50200, Thailand; tayanee.panapimonlawat@gmail.com; 2 Graduate School, Chiang Mai University, Chiang Mai 50200, Thailand; 3 Center of Excellence in Materials Science and Technology, Chiang Mai University, Chiang Mai 50200, Thailand; sphanichphant@gmail.com

**Keywords:** conducting polymer, poly(3-aminobenzylamine), dopamine, layer-by-layer self-assembly, biosensor

## Abstract

Dopamine (DA) is an important neurotransmitter which indicates the risk of several neurological diseases. The selective determination with low detection limit is necessary for early diagnosis and prevention of neurological diseases associated with abnormal concentration of DA. The purpose of this study is to fabricate a poly(3-aminobenzylamine)/poly(sodium 4-styrenesulfonate) (PABA/PSS) multilayer thin film for use as an electrochemical DA biosensor. The PABA was firstly synthesized using a chemical oxidation method of 3-aminobenzylamine (ABA) monomer with ammonium persulfate (APS) as an oxidant. For electrochemical biosensor, the PABA/PSS thin film was fabricated on fluorine doped tin oxide (FTO)-coated glass substrate using the layer-by-layer (LBL) self-assembly method. The optimized number of bilayers was achieved using SEM and cyclic voltammetry (CV) results. The electroactivity of the optimized LBL thin film toward detection of DA in neutral solution was studied by CV and amperometry. The PABA/PSS thin film showed good sensitivity for DA sensing with sensitivity of 6.922 nA·cm^−2^·µM^−1^ and linear range of 0.1–1.0 µM (R^2^ = 0.9934), with low detection limit of 0.0628 µM, long-term stability and good reproducibility. In addition, the selectivity of the PABA/PSS thin film for detection of DA under the common interferences (i.e., ascorbic acid, uric acid and glucose) was also presented. The prepared PABA/PSS thin film showed the powerful efficiency for future use as DA biosensor in real sample analysis.

## 1. Introduction

Dopamine (3,4-dihydroxyphenylethylamine, DA) is one of the key neurotransmitters in the central nervous system of mammals. DA levels affect the human brain functions in transmitting electrical signals, hormonal, emotional, metabolic, renal and also play an important role in immune and cardiovascular systems [1,2]. The normal DA concentration in human blood is in the range 0.01–1 µM [3,4]. The extremely low levels of dopamine increase the risk of neurological diseases such as Parkinson’s disease, Schizophrenia, Tourette’s syndrome, Alzheimer’s disease, Huntington’s disease, hypertension and attention deficit hyperactivity disorder [4,5,6]. It is; therefore, necessary to detect DA in low concentrations. In recent years, several analytical techniques have been proposed to measure the amount of DA in humans, including fluorescence [7], calorimetry [8], liquid chromatography-mass spectrometry [9], high performance liquid chromatography-chemiluminescence [10], and capillary electrophoresis [11]. In addition, the electrochemical techniques (i.e., voltammetry, amperometry, potentiometry and electrochemical impedance spectroscopy) are the interesting alternative methods for electrochemical biosensor applications due to them being facile and low cost and having a short operation time, high sensitivity and low detection limit methods [12, 13, 14, 15]. The graphene oxide-based molecular imprinted polymer (GO-MIP) was prepared for electrochemical cholesterol biosensor using cyclic voltammetry (CV). The obtained biosensor demonstrated high sensitivity, low limit of detection and fast response time [16]. In addition, the analytical performance for the DA detection can be improved using conducting polymer-based biosensors. The poly(pyrrole-3-carboxylic acid) (p(P3CA))-modified pencil graphite electrode (PGE) was developed as a disposable and low-cost electrochemical DA sensor [17].

Conducting polymers have received great attention for electrochemical applications because the π-bond delocalized electrons on the conjugated structures lead to excellent electrical and conductive properties [18,19]. The common conducting polymers for electrochemical biosensors are polyaniline (PANi) [20], polypyrrole (PPy) [21], poly(3,4-ethylenedioxythiophene) (PEDOT) [22] and polythiophene (PTH) [23]. The electrochemical sensor based on PPy/carbon-coated mesoporous silica (PPy/C#SiO_2_) nanocomposite-modified glassy carbon electrodes (GCEs) demonstrated highly specific DA detection in the presence of the interference species [24]. In addition, PEDOT/PANi-modified GCEs was successfully prepared for simultaneous determination of DA, uric acid (UA) and ascorbic acid (AA) [25]. Among the common conducting polymers, PANi and its derivatives are the most popular conducting polymers in electroanalytical studies, which provide high sensitivity, selectivity, conductivity and good stability for detection of biomolecules [15,26]. In addition, they are high surface area materials with rapid response time for sensor applications [27,28]. Several recent studies had been reported on the fabrication of PANi-based electrochemical sensors, for example, PANi-graphene oxide (PANi-GO) fibrous nanocomposites modified GCEs were prepared for simultaneous detection of AA, DA and UA with high selectivity and sensitivity [29]. The fabrication of gold nanoparticles (AuNPs)-PANi composites were developed as a DA sensor with improved catalytic activity [30]. Moreover, nanocomposites of MoS_2_-PANi and reduced graphene oxide (MoS_2_-PANi/rGO) were employed for simultaneous detection of AA, DA and UA with high electrochemical activity [31].

The use of the layer-by-layer (LBL) self-assembly method has attracted widespread attention for the fabrication of electrochemical biosensors. The obtained biosensor films are composed of alternative layers of oppositely charged species, containing positive and negative charges from polyelectrolytes, by electrostatic interaction. The significant advantage of LBL self-assembly method is the number of layers and film thickness are precisely controlled. In addition, this method requires a small quantity of materials [32,33]. The fabrication of PANi-AuNPs composite multilayers through the LBL method was studied for simultaneous detection of DA and UA. The fabricated PANi-AuNPs LBL films showed good electroactivity and high conductivity under neutral solution condition [34]. The fabrication of PANi nanoparticles-carboxylic acid-functionalized multiwalled carbon nanotubes (MWCNTs-COOH) film through the LBL self-assembly method for nifedipine (NIF) sensing has also been recently reported. The obtained LBL films exhibited uniform distribution of PANi-MWCNTs-COOH nanocomposites, which led to electronic communications of active materials and provided high facility, sensitivity and selectivity for detection of biomaterials [35]. 

**Figure 1:**
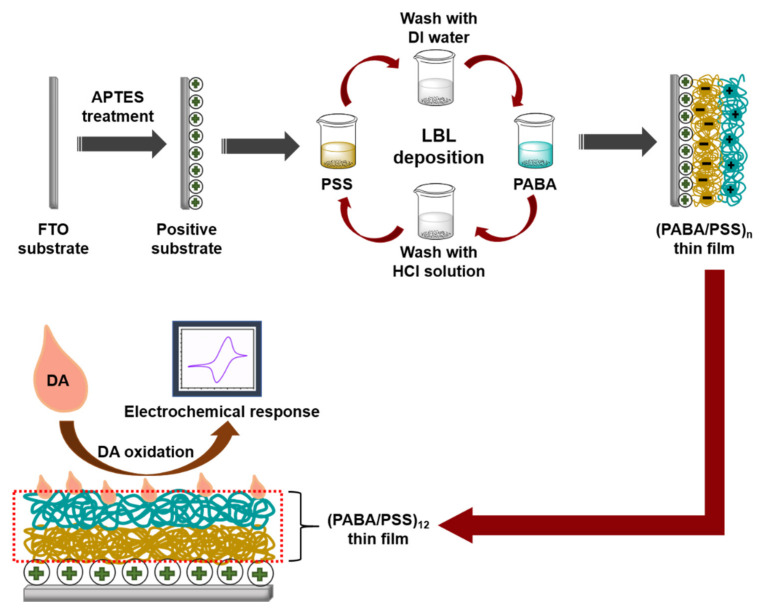
Schematic diagram representing the poly(3-aminobenzyl amine) (PABA)/ poly(sodium 4-styrenesulfonate) (PSS) layer-by-layer (LBL) film assembled for the electrochemical dopamine (DA) biosensor on fluorine doped tin oxide (FTO) substrate

Although various studies have been reported on electrochemical DA biosensors based on polyaniline derivatives or LBL self-assembly, to our knowledge, the development of an electrochemical DA biosensor based on the LBL self-assembly of conductive poly(3-aminobenzylamine) has never been reported. Hence, this study aims to fabricate the PANi derivative (i.e., poly(3-aminobenzylamine) (PABA) thin film) using the LBL method for use as an electrochemical DA biosensor. We have successfully developed the DA biosensor based on the LBL of conductive layers with a low limit of detection. The PABA was synthesized by a chemical oxidation method of 3-aminobenzylamine (ABA) monomer. The LBL multilayer thin film of the synthesized PABA and poly(sodium 4-styrenesulfonate) (PSS) (PABA/PSS) was built up on a fluorine doped tin oxide (FTO)-coated glass substrate, as shown in Figure 1. The obtained thin film was characterized by attenuated total reflectance-Fourier transform infrared (ATR-FTIR) spectroscopy and scanning electron microscopy (SEM). The optimized number of bilayers was achieved using SEM and CV results. The optimized PABA thin film (12 bilayers) was used as a working electrode for electrochemical detection of DA. The electrochemical activity in neutral solution of the obtained electrode was studied by CV and amperometry techniques. The PABA thin film exhibits a good electrochemical performance towards DA detection. The prepared PABA/PSS thin film showed powerful efficiency for future use as DA biosensor in real sample analysis.

## 2. Experimental

### 2.1. Chemicals and Materials

Poly(sodium 4-styrenesulfonate) (PSS; average Mw ⁓70000, 30 wt.% in H_2_O), 3-aminobenzyl amine (ABA), (3-aminopropyl)triethoxysilane (APTES), 4-(2-hydroxyethyl) piperazine-1-ethanesulfonic acid (HEPES) and dopamine (DA) were purchased from Sigma-Aldrich (Darmstadt, Germany). Ammonium persulfate (APS), toluene, ethanol and methanol were pur-chased from RCI Labscan (Bangkok, Thailand). Ascorbic acid (AA) was purchased from Poch (Gliwice, Poland). Uric acid (UA) was purchased from Bio Basic (Markham, ON, Canada). Potassium hydroxide (KOH) and hydrochloric acid (HCl) were purchased from QRëC™ (Auckland, New Zealand). All chemicals were used as received without further puri-fication. All solutions were prepared with deionized (DI) water. Fluorine-doped tin oxide (FTO) coated glass substrate was purchased from Sigma-Aldrich and cleaned prior to employing for the working electrode.

### 2.2. Synthesis of PABA and Fabrication of PABA/PSS Thin Films

PABA was synthesized by chemical oxidation of ABA monomer using APS as an oxidant. A 50 mM ABA aqueous solution was magnetically stirred for 15 min. APS (50 mM) was gradually added into ABA solution under continuous stirring for 1 h. The obtained mixture was purified through alkaline precipitation by adding 10% KOH solution until pH of the mixture reached 10 [36]. The mixture was centrifuged at 6000 rpm for 10 min, rinsing with DI water and methanol, respectively. The PABA stock solution was prepared by dissolving the precipitated PABA (0.1% *w*/*v*) in 0.5 M HCl. The PABA solution was sonicated for 15 min before use for fabrication of the PABA/PSS thin film. The PSS solution was prepared by dissolving PSS (0.33% *v*/*v*) in DI water. The PABA/PSS thin film was fabricated by LBL self-assembly method. The FTO electrode was firstly treated with APTES at 96 °C for 2 h and immersed into 0.1 M HCl overnight [37]. For LBL deposition, the FTO electrode was then immersed into PSS solution for 15 min and washed with DI water for 1 min, followed by dipping into PABA solution for 15 min and washed with 0.5 M HCl for 1 min. This LBL deposition was repeated until the desired number (n) of bilayers was obtained as represented in Figure 1. The fabricated PABA/PSS thin film was employed for electrochemical DA biosensor.

### 2.3. Materials Characterizations

The chemical constituents of the synthesized PABA were investigated using ATR-FTIR spectroscopy (Bruker Tensor 27, Billerica, MA, USA) and proton nuclear magnetic resonance (^1^H-NMR) spectroscopy (Bruker 400 MHz, Ettlingen, Germany). The optical properties of the synthesized PABA were studied using UV–Vis absorption spectroscopy (UV-1800 Shimadzu, Tokyo, Japan) and photoluminescence (PL) spectroscopy (Synergy™ H4, Winooski, VT, USA). The thermal properties were characterized by differential scanning calorimetry (DSC; Mettler-Toledo, Columbus, OH, USA) and thermogravimetry analysis (TGA; Rigaku Thermo plus EVO2 TG-DTA 8122 Akishima, Japan). The cross section and top view morphologies of the PABA/PSS thin film were performed using a field emission-scanning electron microscopy (FE-SEM; JEOL JSM-6335F Tokyo, Japan).

### 2.4. Electrochemical Detection of DA

All electrochemical experiments were performed using a potentiostat (eDAQ: ED410 e-corder 410, Colorado Springs, CO, USA) with three-electrode system using Ag/AgCl electrode as reference electrode, a platinum wire as counter electrode and FTO as working electrode. The optimized number of PABA/PSS bilayer for use as electrochemical DA biosensor was investigated by CV measurement in 0.5 M HCl solution under applied potential range of −0.2 to 0.8 V at scan rate of 400 mV/s. The CV of the optimized PABA/PSS thin film at various scan rates (20–300 mV/s) was studied using the potential range of −0.2 to 0.7 V in 10 mM HEPES buffer (pH 7.0) containing 5 mM K_3_Fe(CN)_6_ and 0.1 M KCl. The sensing examination of the optimized PABA/PSS biosensor was performed using CV with potential range of −0.2 to 0.7 V at a scan rate of 100 mV/s in the buffer toward a concentration of 1 mM DA. In addition, the amperometric responses were obtained by successive addition of various concentrations of DA (0.01 nM to 10 mM) into the buffer at constant applied potential of 0.3 V. For the selectivity experiment, CV was employed for DA detection in the presence of AA, UA and glucose with a potential range of −0.2 to 0.7 V at a scan rate of 100 mV/s in the buffer. The amperometry was further performed in the buffer at constant applied potential of 0.3 V to evaluate the long-term stability of the biosensor electrodes for 30 days.

## 3. Results and Discussion

### 3.1. Characterization of PABA

**Figure 2:**
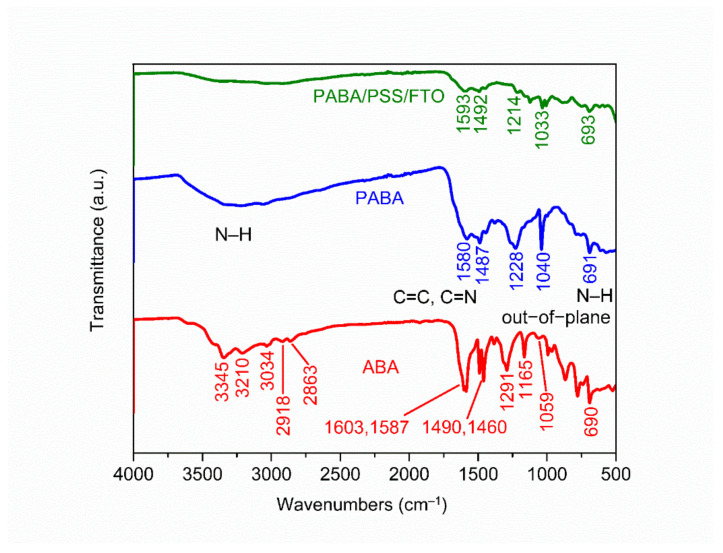
Attenuated total reflectance-Fourier transform infrared (ATR-FTIR) spectra of the 3- aminobenzyl amine (ABA) monomer, synthesized PABA and the PABA/PSS LBL film on FTO.

The chemical constituent of the ABA monomer, synthesized PABA and PABA/PSS LBL thin film on FTO substrate was characterized by ATR-FTIR. The ATR-FTIR spectra in the range of 500–4000 cm^−1^ of ABA, PABA and PABA/PSS thin film are shown in Figure 2. The asymmetric and symmetric N–H stretching of amine groups in the ABA structure showed the absorption peaks at 3345 and 3210 cm^−1^, respectively [38]. The =C–H stretching of the aromatic ring represented the peak at 3034 cm^−1^. The presence of peaks at 2918 and 2863 cm^−1^ indicated asymmetric and symmetric C–H stretching of alkane groups in the ABA structure, respectively [39]. Meanwhile, the synthesized PABA showed the absorption band at around 3000–3500 cm^−1^ which related to the presence of N–H stretching of amine groups, =C–H stretching of aromatic rings and C–H stretching of alkane groups on the PABA chain [40]. The C=C stretching of aromatic rings for ABA showed the peaks at 1603 and 1587 cm^−1^, while PABA showed the peak at 1487 cm^−1^. The C=C and C=N stretching of quinoid rings for PABA exhibited the significant peak at 1580 cm^−1^, which indicated that the synthesized PABA was composed of amine units [41,42]. The other significant peaks at 1291 and 1059 cm^−1^ for ABA and 1228 and 1040 cm^−1^ for PABA corresponded to C–N stretching of the aromatic ring and aliphatic amine, respectively [38,43]. In addition, the –NH_2_ out-of-plane bending vibration for ABA and PABA showed similar peaks at 690 and 691 cm^−1^, respectively [38,44]. It indicated that PABA could be successfully synthesized using the ABA monomer. The ATR-FTIR spectrum of the PABA/PSS thin film was approximately identical to the PABA, which confirms the formation of the PABA/PSS LBL multilayer thin film on the FTO substrate.

The common characterization technique for confirmation of the structure of the synthesized material is ^1^H-NMR spectroscopy. The ^1^H-NMR analysis of the ABA monomer and PABA (Figure S1 of Information) was carried out using deuterium oxide (D_2_O) as a solvent. The ^1^H-NMR spectrum of the ABA monomer presented three main signals (a,b–d,e) with the D_2_O signal at 4.70 ppm. The signal at 3.52 ppm corresponded to the methylene protons (a), which attached to the benzene ring of ABA. The multiplet signal at 6.59–6.66 ppm was attributed to aromatic protons (b–d), which were adjacent to the amine and methylene groups. The triplet signal at 7.05–7.08 ppm contributed to the characteristic signal of the aromatic proton (e). The ^1^H-NMR spectrum of PABA showed two main signals with the broad D_2_O signal at 4–5.5 ppm. The methylene proton signal appeared at 3.34 ppm (a) and the aromatic protons attached to the benzenoid and quinoid rings of PABA showed the multiplet signals 6.55–7.31 ppm (b–i). It could be concluded that the synthesis of PABA from the ABA monomer using APS as an oxidant was successful in this study [36,45].

The optical properties of the ABA monomer and the PABA were investigated by UV–Vis absorption spectroscopy and photoluminescence spectroscopy. The UV–Vis absorption spectra of the ABA monomer in DI water and PABA in 0.5 M HCl are shown in Figure S2 of the Supplementary Information. The absorption peaks at 232 and 285 nm were assigned for the π–π* transition, which represented the presence of aniline moiety and benzene rings, respectively. The PABA in 0.5 M HCl showed two main absorption peaks at 240 and 300 nm. The peak at 240 nm was assigned to the π–π* transition within the aniline moiety, whereas the peak at 300 nm was assigned to the π–π* transition attributed to benzenoid rings and lone pair electrons of nitrogen [40,46]. In addition, the PABA exhibited a broad absorption band around 500 nm, which was attributed to the excitonic π–π* transition from the highest occupied molecular orbital (HOMO) of the valence band to the lowest unoccupied molecular orbital (LUMO) of the conduction band [36,40,47]. For PL measurement, the ABA monomer and PABA solutions were excited in the range of 340–380 nm (Figure S3 of Supplementary Information). The PL intensity decreased with increasing excitation wavelength. The ABA and PABA exhibited PL signals with peak maxima at 446 nm, which were due to the π–π* transition of the benzenoid units. The PL spectra of PABA showed a further broad peak at 530 nm, which implied the de-excitation of the polymer backbone and the effect of the chlorine ion dopant from the HCl solution [43,46,48,49].

The thermal behaviors of the synthesized PABA were analyzed by DSC and TGA techniques (Figure S4 of Supplementary Information). The DSC curve illustrates both endothermic and exothermic thermal peaks during the temperature scan from 0 to 400 °C. The first endothermic transition was glass transition temperature (T_g_) at 75 °C, which corresponded to physical change, such as the loss of adsorbed water in the form of moisture, and chemical change, such as the absorption of energy for decomposition [50]. The other endothermic transitions at 148 and 322 °C may be attributed to the melting point (T_m_) [51] and degradation point (T_d_) of the polymer backbone, respectively [50]. The TGA thermogram of the obtained PABA at a heating rate of 10 °C/min from 25–475 °C in nitrogen exhibited two major stages of weight loss (i.e., at temperatures below 100 °C and above 250 °C). At the temperature below 100 °C, the weight loss was 10%, which was attributed to the expulsion of water contained in the PABA residue [52]. The PABA showed thermal stability up to 250 °C. The weight loss of PABA was represented again at above 250 °C, which was related to the structural decomposition of the polymer chain [53].

### 3.2. Fabrication of PABA/PSS Film

**Figure 3:**
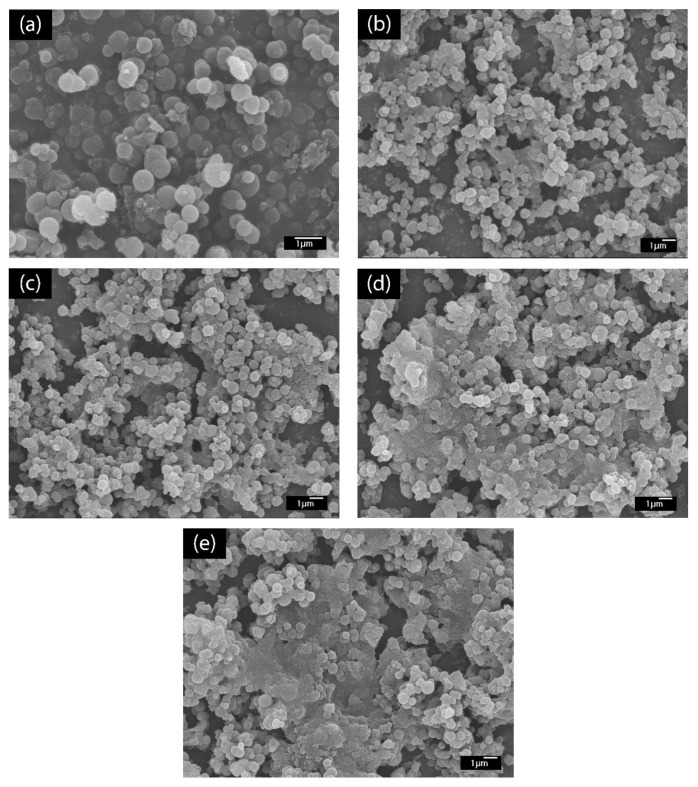
SEM images (top view) of (**a**) synthesized PABA powder and (**b**) 10-, (**c**) 12-, (**d**) 14- and (**e**) 20-bilayer PABA/PSS thin films.

The LBL multilayer thin film of PABA/PSS was built up on the FTO-coated glass substrate, as shown in Figure 1. The electrostatic interaction of the positively-charged PABA (–NH_3_^+^) and negatively-charged PSS (–SO_3_^−^) resulted in the formation of the stable LBL multilayer thin film of PABA/PSS (Supplementary Information, Figure S5). The morphology of the synthesized PABA and PABA/PSS thin film was investigated by SEM technique, as shown in Figure 3. The SEM image of the PABA synthesized via the chemical oxidation reaction exhibited identical and spherical shapes, with an average diameter of about 0.65 µm, as represented in Figure 3a. The synthesized PABA was employed for fabrication of the multilayer thin film in combination with PSS on FTO substrate through the LBL self-assembly technique. The optimum number of PABA/PSS bilayer films was evaluated from SEM images and electric charge (Q) analysis. The SEM images of the fabricated PABA/PSS thin film with 10, 12, 14 and 20 bilayers are shown in Figure 3b–e, respectively. The morphology and the packing of a conducting polymer material significantly affect the performance of the conducting polymer-assembled electrodes. Therefore, the agglomeration of a denser PABA/PSS film could affect the effective surface area and porosity [54]. The 10-bilayer PABA/PSS thin film was a sparsely distributed film on the surface, as seen in Figure 3b, whereas the 12-bilayer thin film was densely packed, as observed in Figure 3c. However, the films exhibited agglomeration between PABA and PSS layers when the number of bilayers was increased to 14 and 20, as represented in Figure 3d,e, respectively. This agglomeration could hinder redox processes of the obtained film for electrochemical DA detection. It could be concluded from SEM results that the 12-bilayer PABA/PSS thin film was the optimized film composition for this study, which corresponded to the electrical performance result that will be discussed further.

**Figure 4:**
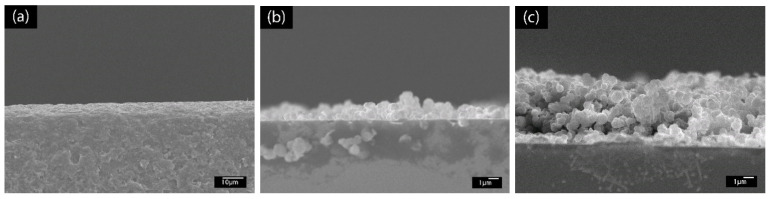
SEM images (cross sectional view) of (**a**) glass substrate, (**b**) one- and (**c**) 12-bilayer PABA/PSS thin films.

To further confirm the buildup of the PABA/PSS LBL multilayer thin film, SEM images in cross sectional view were examined, as shown in Figure 4. Figure 4a shows the SEM image of the bare glass substrate without PABA/PSS thin film. After deposition of PABA/PSS thin film on the substrate through the LBL self-assembly method, the bilayers of the PABA/PSS thin film were observed. The SEM image of one bilayer is shown in Figure 4b with the average thickness of 89.34 µm. Figure 4c shows the SEM cross sectional view of the 12-bilayer PABA/PSS thin film with the average thickness of 379.17 µm. It can be seen that the thickness of the PABA/PSS thin film was increased from one bilayer to 12 bilayers due to the progressive loading of PABA and PSS layers, which was composed of alternating layers of oppositely charged species by electrostatic interaction. This indicates that a good deposition of thin film on the substrate surface can be achieved.

**Figure 5:**
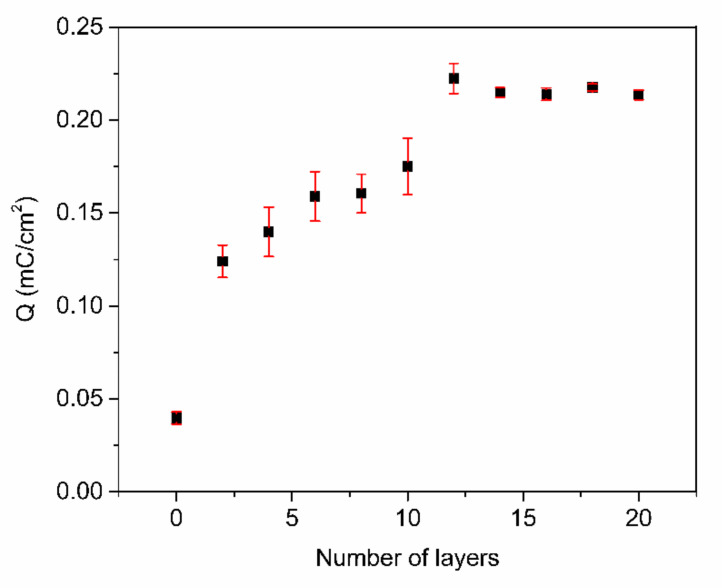
The electric charge (Q) of the PABA/PSS film with an increasing number of bilayers, obtained from CV measurement in the potential range of −0.2 to 0.8 V at a scan rate of 0.4 V/s in 0.5 M HCl.

To further optimize the number of PABA/PSS bilayers prior to employment for fabrication of the electrochemical DA biosensor, the PABA/PSS thin film was fabricated on an FTO-coated glass substrate using the LBL self-assembly method. The electric charge of the different numbers of PABA/PSS bilayer films was investigated by CV measurement in 0.5 M HCl, under an applied potential range of −0.2 to 0.8 V at a scan rate of 0.4 V/s for one cycle. As seen in Figure 5, the electric charge increased with the number of bilayers due to the progressive loading of PABA and PSS bilayers. The maximum electric charge of the assembled bilayer was 12 bilayers, which was the optimum number for this study. The electric charge showed a negligible increase after 12 bilayers, which may be the effect of agglomeration which could hinder the redox processes of the obtained film in the electrochemical process [54]. Meanwhile, the agglomeration of a denser PABA/PSS film could affect the effective surface area and porosity, which lead to a decrease of specific catalytic surface area. This electrical performance data were consistent with the SEM analysis. 

**Figure 6:**
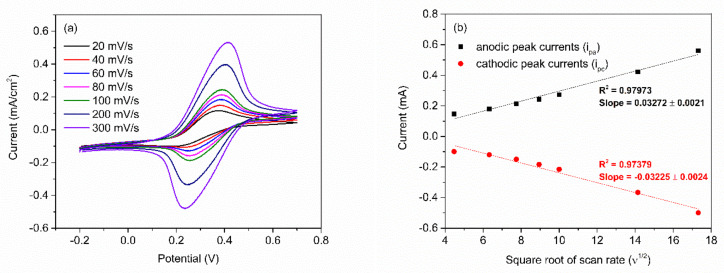
(**a**) CV responses of the 12-bilayer PABA/PSS electrode at different rates in 10 mM HEPES buffer (pH 7.0), containing redox mediator (5 mM K_3_Fe(CN)_6_ and 0.1 M KCl); and (**b**) the linear relationship of anodic and cathodic peak currents as a function of the square root of scan rates (ν^1/2^).

After obtaining the optimum number of PABA/PSS bilayers, the electrochemical characteristic of the 12-bilayer PABA/PSS electrode was examined through CV in the potential range of –0.2 to 0.7 V, using K_3_Fe(CN)_6_ solution as the redox mediator. The electrochemical response of the 12-bilayer PABA/PSS thin film at different scan rates (20–300 mV/s) in 10 mM HEPES buffer (pH 7.0), containing 5 mM K_3_Fe(CN)_6_ and 0.1 M KCl, was studied, as shown in Figure 6. The oxidation and reduction currents were increased with increasing of the scan rate. The CV responses provided the average difference between anodic and cathodic peak voltage (∆E_a,c_) of ~142 mV. The obtained ∆E_a,c_ were rather broad redox peaks, which indicated that the PABA/PSS electrode exhibits quasireversible electron transfer process as seen in Figure 6a. In addition, the oxidation and reduction currents were linearly proportional to the square root of the scan rate (ν^1/2^), as presented in Figure 6b, which indicated the linear diffusion-controlled at the electrode surface [55].

### 3.3. Electrochemical Detection of DA

**Figure 7:**
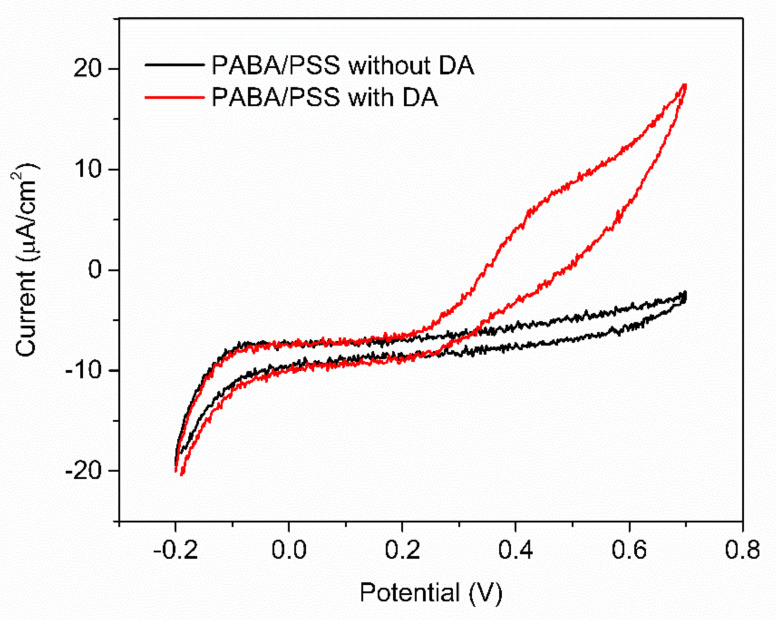
CV responses of the 12-bilayer PABA/PSS electrode at a scan rate of 100 mV/s in 10 mM HEPES buffer of 1 mM DA.

**Figure 8:**
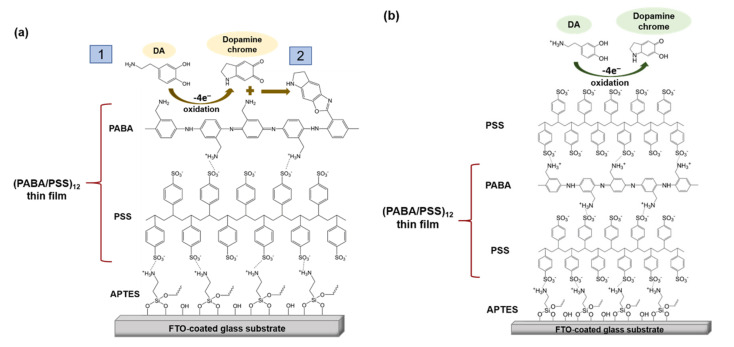
Proposed electrochemical oxidation mechanism for dopamine on the PABA/PSS electrode surface in neutral pH solution (**a**) PABA and (**b**) PSS as the last layer.

**Figure 9:**
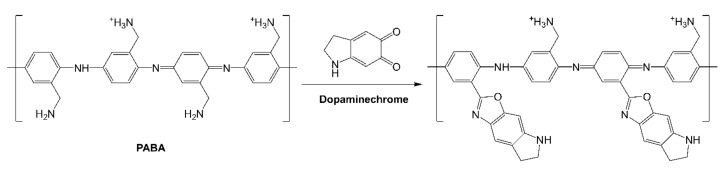
Proposed possible covalent bond formation between PABA and oxidized dopamine or dopaminechrome.

The obtained 12-bilayer PABA/PSS thin film was employed for electrochemical DA biosensor. The electrochemical performances, including sensitivity, selectivity, reproducibility and stability of the PABA/PSS biosensor, were studied towards DA detection using CV and amperometry techniques. The electrocatalytic activity of the optimized PABA/PSS biosensor was performed, using CV with a potential range of −0.2 to +0.7 V at a scan rate of 100 mV/s in 10 mM HEPES buffer toward 1 mM DA, for one cycle. The CV responses of the 12-bilayer PABA/PSS biosensor with and without DA are shown in Figure 7. The oxidation current response at 0.4 V was observed in the presence of 1 mM DA, while a negligible current was observed without DA. In addition, to examine the usefulness of the obtained 12-bilayer PABA/PSS thin film, the electrocatalytic activities of the obtained 12-bilayer PABA/PSS film and bare FTO substrate were also investigated using CV in the presence of 1 mM DA in neutral solution (Supplementary Information, Figure S6). The current response of the obtained 12-bilayer PABA/PSS film was obviously higher than that of bare FTO. It implied that the electro-oxidation current response of the modified electrode was due to the PANi derivative could catalyze the DA oxidation, and also exhibited the electron transfer kinetics for the DA redox [24,56]. At the surface of PABA/PSS electrode, with PABA as the last layer, as shown in Figure 8a, the improvement of electrocatalytic activity could be probably due to the π–π interaction of the phenyl structures of DA and electroactive PABA [29]. DA in neutral form at neutral pH is firstly electrochemically oxidized to o-dopaminoquinone with a two-electron transfer process on the PABA/PSS electrode, which then leads to the cyclization into leukodopaminechrome in neutral pH solution [57]. The leukodopaminechrome can be further oxidized with a two-electron transfer process to dopaminechrome [57] that can form covalent bonds with PABA chains on the electrode surface [58]. The carbonyl group of oxidized dopamine or dopaminechrome can form strong covalent bonds with the amine group of PABA on the electrode, as illustrated in Figure 9 [58]. In contrast, if the non-electroactive polyelectrolyte (PSS) is located at the last layer, as shown in Figure 8b, the oxidation process of positively-charged DA would be slowly exhibited, because the non-electrochemical connection of PSS could block the interaction of DA with the electroactive PABA [36]. Furthermore, the dopaminechrome in this DA oxidation process could react with other DA molecules, which leads to the electrode fouling effect [59].

**Figure 10:**
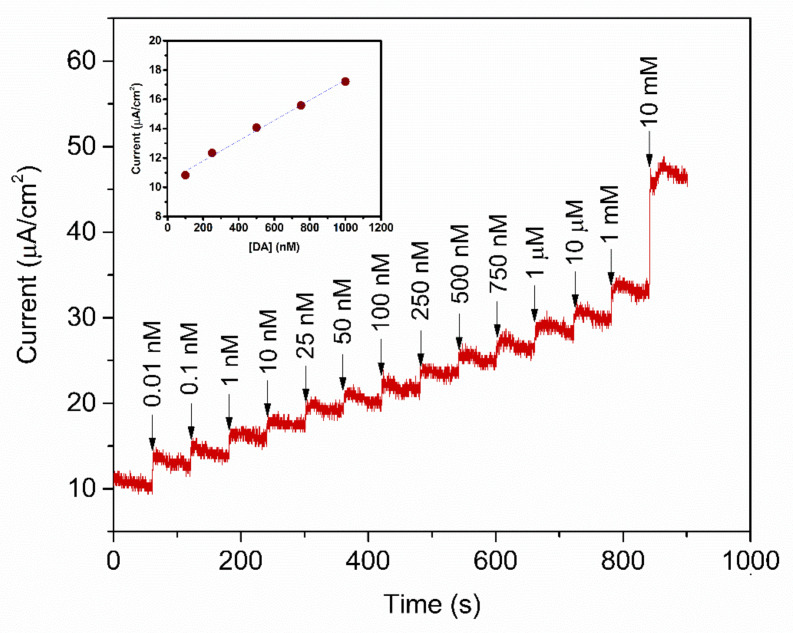
Amperometric response of the 12-bilayer PABA/PSS electrode at 0.3 V (vs. Ag/AgCl) in 10 mM HEPES buffer with successive addition of various concentrations of DA. Inset: Corresponding calibration curve for DA detection.

To study the analytical performance, the amperometric measurement of the fabricated PABA/PSS biosensor electrodes was carried out, for detection of various concentrations of DA, at the constant applied potential of 0.3 V versus Ag/AgCl in 10 mM HEPES buffer. As represented in Figure 10, the amperometric current response increased with successive addition of different concentrations of DA, from 0.01 nM to 10 mM. The corresponding calibration curve of the PABA/PSS biosensor presented the linear range from 0.1 to 1.0 µM, with a correlation coefficient of 0.9934, as shown in the inset of Figure 10. The sensing performance with good sensitivity of 6.922 nA·cm^−2^·µM^−1^ was obtained from the slope of this calibration curve, whereas the low limit of detection (LOD) of 0.0628 µM can be; therefore, calculated from the slope of this obtained calibration curve and standard deviation of amperometric responses of HEPES blank solution [60]. The comparison of the developed PABA/PSS electrodes with some previous reports of the electrochemical biosensors based on PANi and its derivatives, for the detection of DA, was accumulated in Table 1. The developed PABA/PSS biosensors exhibited the lower limit of detection and linear range. As we mentioned in the introduction part about the normal DA concentration in human blood, it; therefore, could be concluded that the fabricated PABA/PSS electrodes would be suitable for DA detection in real samples in the future.

**Figure 11:**
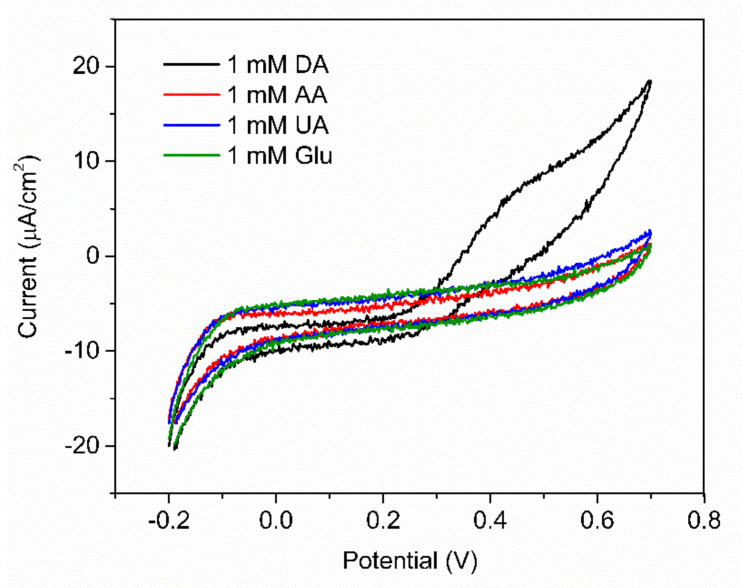
CV responses of the 12−bilayer PABA/PSS electrode with the addition of 1 mM DA, ascorbic acid (AA), uric acid (UA) and glucose at a scan rate 100 mV/s in 10 mM HEPES buffer

In order to evaluate the selectivity of the fabricated PABA/PSS electrode for DA sensing with the common interferences, the anti-interference property of the electrode was investigated using CV with addition of 1.0 mM DA, glucose, UA and AA. Glucose is usually tested as an interfering species because the human serum glucose level is higher than DA, which can normally interfere with the determination of DA. In additon, it is well known that UA is a strong H-bond-forming molecule and may interact with PABA. Furthermore, both UA and AA can be oxidized at similar potentials to DA [58,66]. As shown in Figure 11, the current response was obviously observed upon addition of 1.0 mM DA, whereas negligible current responses were found after addition of the interfering species. This result implied that the developed PABA/PSS biosensor electrode exhibited the anti-interference property in the presence of glucose, UA and AA with good selectivity for DA detection. The common interferences for the electrochemical dopamine sensor are mainly AA and UA, which can be oxidized at similar potentials to DA. In addition, other catecholamine neurotransmitters, especially tyrosine, L-DOPA, epinephrine (EP) and norepinephrine (NE), may also affect the analysis of DA because their chemical structures are similar to DA. However, due to their functional groups and molecular sizes, these molecules consist of bulk and branched molecules, such as methyl, carboxyl and hydroxyl [67], which may be difficult to introduce successfully onto the PABA/PSS thin film. Therefore, the PABA/PSS modified FTO electrode still plays a key role in confining the responses of the other catecholamine neurotransmitters, because the amine group of PABA can form covalent bonds with the carbonyl group of oxidized dopamine [58].

**Figure 12:**
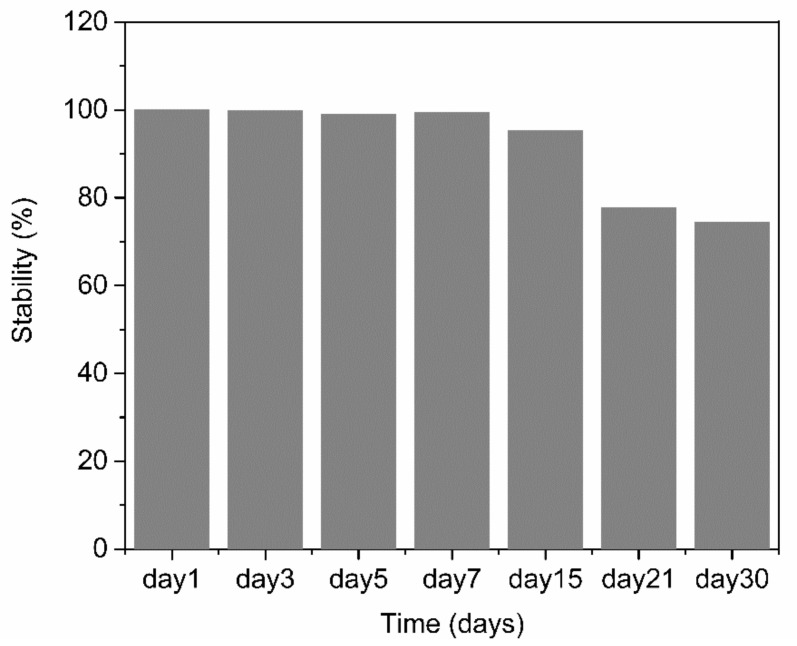
Long-term stability histogram of the 12-bilayer PABA/PSS LBL thin film electrode under electrochemical detection of DA.

The reproducibility and stability of the PABA/PSS biosensor electrode were performed by amperometry. The reproducibility was measured from the current response at 0.3 V versus Ag/AgCl after addition of 1.0 mM DA to the fabricated electrodes. All electrodes displayed consistent current responses with the relative standard deviation (RSD) value of 3.27%, which demonstrated that the fabricated electrodes were acceptable and had excellent reproducibility. The long-term stability of the biosensor electrodes was evaluated by measuring the amperometric responses after every two-day interval for one week and after every seven days or one-week interval for one month. The PABA/PSS biosensor electrodes were stored at room temperature and without humidity for up to 30 days. As shown in Figure 12, the performance of the fabricated electrodes during the first week was almost unchanged compared to the initial response (Day 1). After 30 days, the current response of the PABA/PSS electrodes retained about 74% of the initial response. These results indicated that the fabricated DA biosensor based on the PABA/PSS self-assembled multilayers would be able to detect low levels of DA concentrations with good selectivity and stability, which will be useful for clinical diagnosis applications in the future.

## 4. Conclusions

The electrochemical biosensor based on PABA self-assembled multilayers for the detection of DA was successfully developed using the LBL self-assembly method. For building up the biosensor, the CV measurements showed that the 12 bilayers was the optimum loading number for the PABA/PSS thin film, with maximum electric charge (Q). The SEM result also confirmed this optimized bilayer film. The electrochemical performance of the built up electrode could detect low levels of DA concentrations with a good sensitivity of 6.922 nA·cm^−2^·µM^−1^, in the linear range of 0.1–1.0 µM (R^2^ = 0.9934), with a low detection limit of 0.0628 µM. The DA detection also demonstrates high selectivity in the presence of the common interferences (i.e., glucose, UA and AA). Furthermore, the obtained biosensor showed long-term stability and good reproducibility. This indicates that the developed PABA/PSS biosensor electrode is an effective platform for real diagnosis in the future.

## Figures and Tables

**Table 1:** Electrochemical biosensors based on the PANi-modified electrode for the detection of DA.

Modified Electrode	Linear Range (µM)	Limit of Detection (µM)	Sensitivity	Ref.
GCE/PEDOT/PANi	30–1000	4.58	-	[25]
PANi-GO/GCE	2–18	0.5	2.0 (µA.µM^−1^)	[29]
AuNPs-PANi/GCE	3–115	0.8	0.0269 (µA.µM^−1^)	[30]
MoS_2_PANi/rGO/GCE	5.0–500	0.70	-	[31]
PANi-AuNP multilayers	7–148	3	-	[34]
PANi/CQDs	10–90	0.1013	8.025 (nA·cm^−2^·µM^−1^)	[61]
GCE/PANi-H_2_SO_4_@Au	10–100	6.7	0.045 (µA.µM^−1^)	[62]
Fe_3_O_4_@Au-Cys/PANi/GFE	20–1000	2.19	-	[63]
AuNPs/MoS_2_-PANi composites	1–500	0.1	0.0274 (µA.µM^−1^)	[64]
AuNPs@PANi nanocomposites	10–1700	5	-	[65]
PABA/PSS	0.1–1.0	0.0628	6.922 (nA·cm^−2^·µM^−1^)	This work

Abbreviations: Glassy carbon electrodes (GCE); poly(3,4-ethylenedioxythiophene) (PEDOT); molybdenum disulfide (MoS_2_); reduced graphene oxide (rGO); gold-coated magnetite (Fe_3_O_4_@Au); cysteine (Cys); graphite screen-printed elec-trode (GFE); graphene oxide (GO); gold nanoparticles (AuNPs).

## Supplementary Materials

The following are available online at https://www.mdpi.com/article/10.3390/polym13091488/s1, Figure S1: 1H−NMR spectra of ABA and the synthesized PABA in D2O; Figure S2: UV−Vis absorption spectra of ABA and PABA solutions; Figure S3: PL spectra of (a) ABA and (b) PABA solutions at excitation wavelengths of 340–380 nm; Figure S4: (a) DSC and (b) TGA thermograms of the synthesized PABA; Figure S5: Schematic diagram representing the electrostatic interaction of LBL self−assembled PABA/PSS. Figure S6: DA electro−oxidation on bare FTO and 12−bilayer PABA/PSS/FTO electrode at scan rate 100 mV/s in 10 mM HEPES buffer.
