# Supplementary material for: Electrochemical Dopamine Biosensor Based on Poly(3-aminobenzylamine) Layer-by-Layer Self-Assembled Multilayer Thin Film
Source: Polymers (Basel). 2021 May 6;13(9):1488. doi: 10.3390/polym13091488 (PMC8125673; doi:10.3390/polym13091488)
Supplement: Supplementary file 1 [file polymers-13-01488-s001.zip › polymers-1178729-SI.pdf]

polymers

MDPI

Article

Electrochemical Dopamine Biosensor Based on Poly(3-aminobenzylamine) Layer-by-Layer Self-Assembled Multilayer Thin Film

Tayanee Panapimonlawat [1,2], Sukon Phanichphant [3] and Saengrawee Sriwichai [1,3,*]

[1] Department of Chemistry, Faculty of Science, Chiang Mai University, Chiang Mai 50200, Thailand; tayanee.panapimonlawat@gmail.com
[2] Graduate School, Chiang Mai University, Chiang Mai 50200, Thailand;
[3] Center of Excellence in Materials Science and Technology, Chiang Mai University, Chiang Mai 50200, Thailand; sphanichphant@gmail.com
* Correspondence: saengrawee.s@cmu.ac.th

Citation: Panapimonlawat, T.; Phanichphant, S.; Sriwichai, S. Electrochemical Dopamine Biosensor Based on Poly(3-aminobenzylamine) Layer-by-Layer Self-Assembled Multilayer Thin Film. *Polymers* **2021**, *13*, 1488. https://doi.org/10.3390/polym13091488

Academic Editor: Laura Sola

Received: 26 March 2021
Accepted: 3 May 2021
Published: 6 May 2021

Publisher's Note: MDPI stays neutral with regard to jurisdictional claims in published maps and institutional affiliations.

Copyright: © 2021 by the authors. Licensee MDPI, Basel, Switzerland. This article is an open access article distributed under the terms and conditions of the Creative Commons Attribution (CC BY) license (http://creativecommons.org/licenses/by/4.0/).

Supplementary Figures

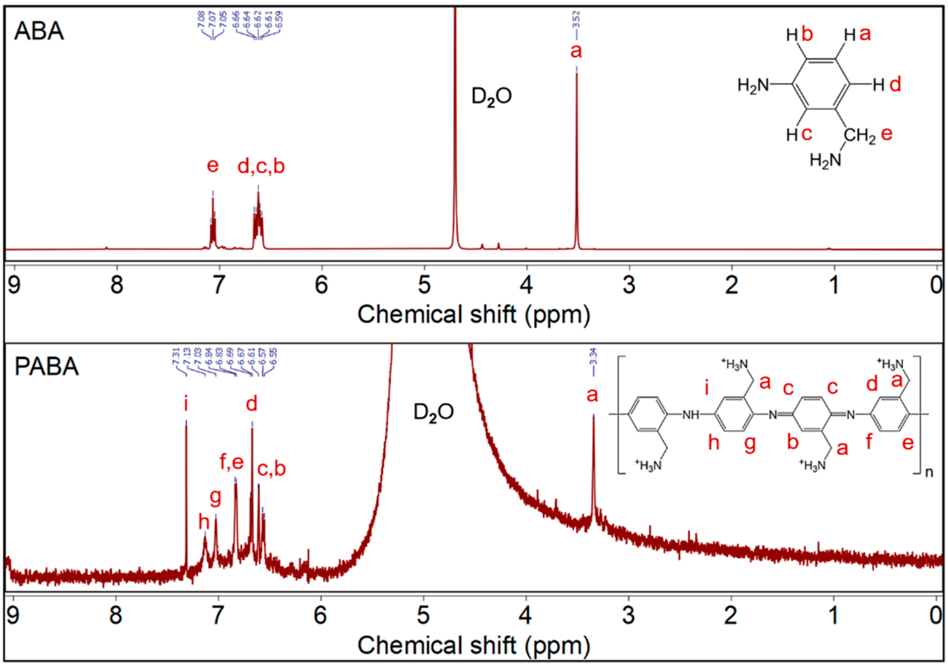

Figure S1. ^{1}H-NMR spectra of ABA and the synthesized PABA in D_2O.

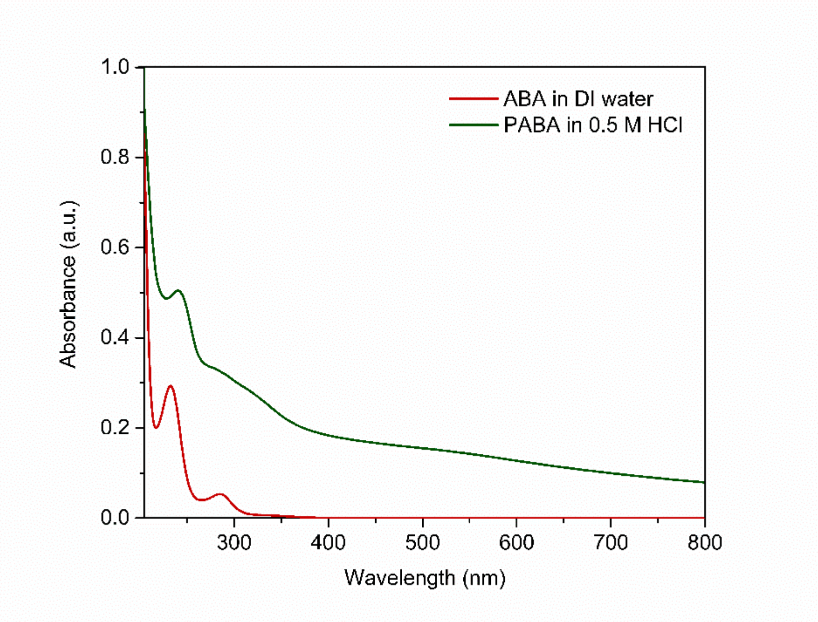

Figure S2. UV-Vis absorption spectra of ABA and PABA solutions.

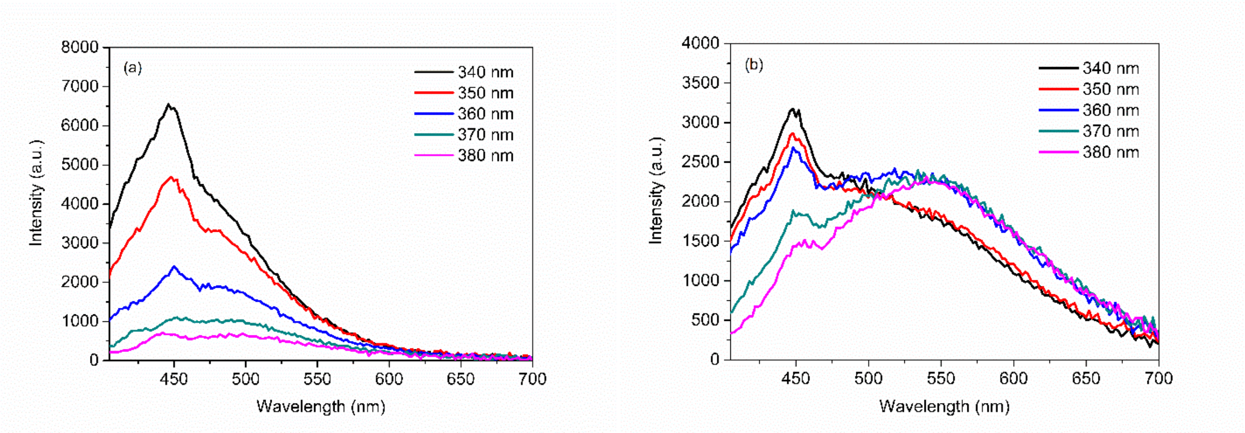

Figure S3. PL spectra of (**a**) ABA and (**b**) PABA solutions at excitation wavelengths of 340–380 nm.

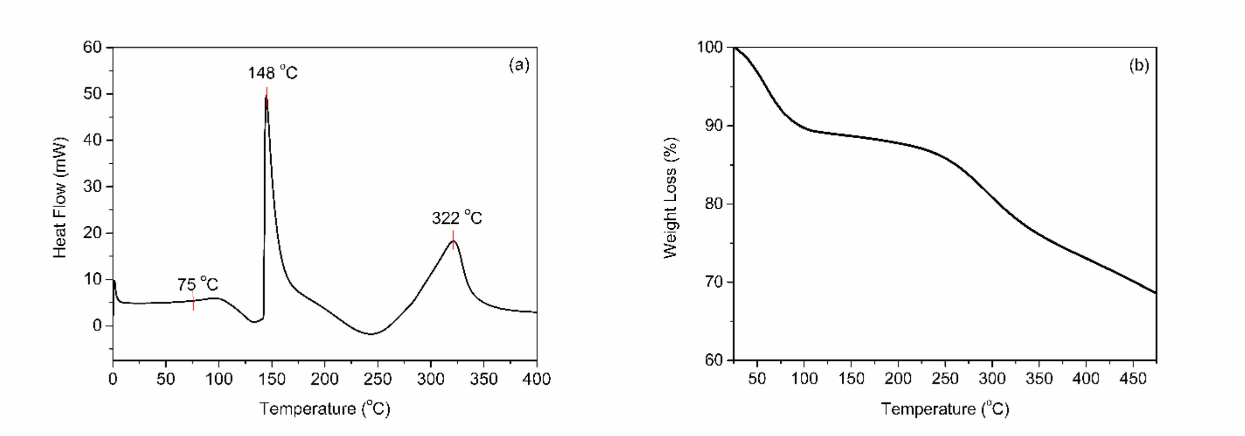

Figure S4. (**a**) DSC and (**b**) TGA thermograms of the synthesized PABA.

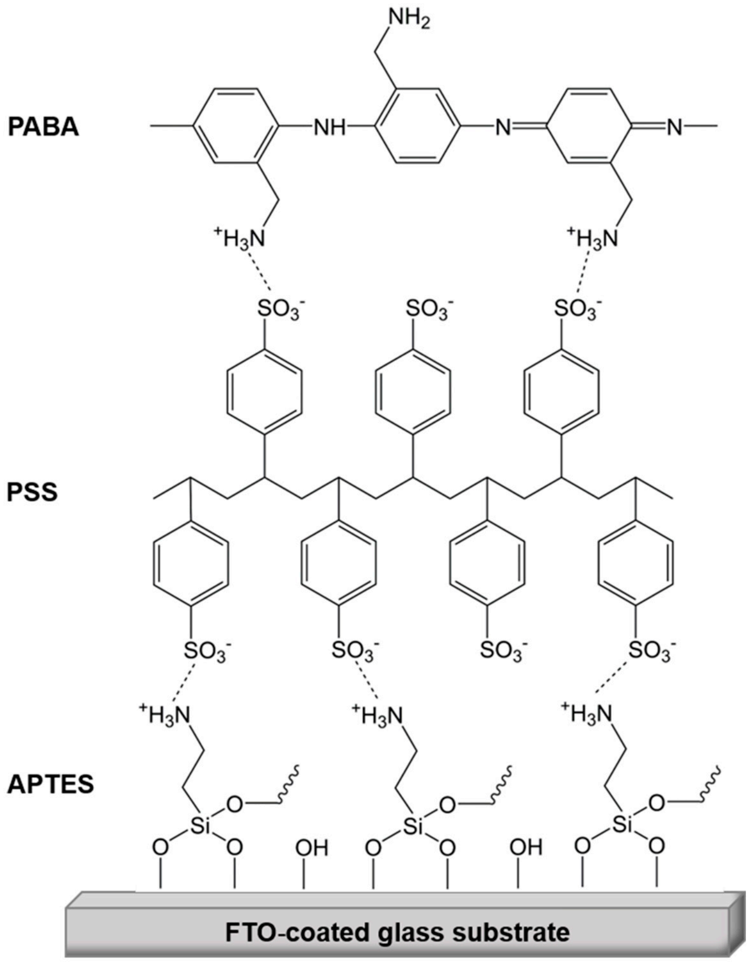

Figure S5. Schematic diagram representing the electrostatic interaction of LBL self-assembled PABA/PSS.

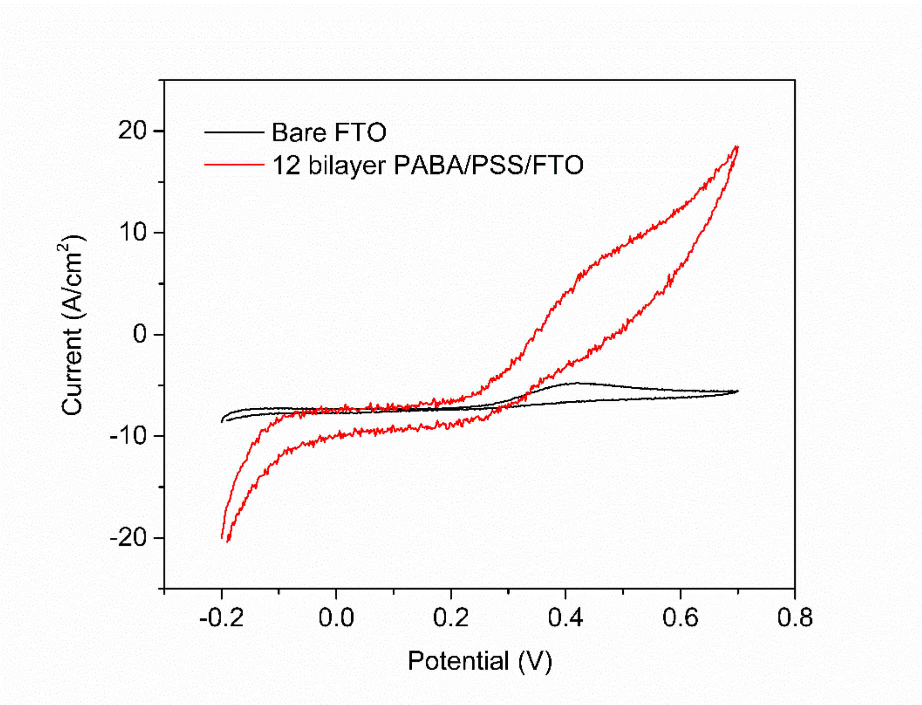

Figure S6. DA electro-oxidation on bare FTO and 12 bilayer PABA/PSS/FTO electrode at scan rate 100 mV/s in 10 mM HEPES buffer.
